# Correlation between national surveillance and search engine query data on respiratory syncytial virus infections in Japan

**DOI:** 10.1186/s12889-022-13899-y

**Published:** 2022-08-09

**Authors:** Kazuhiro Uda, Hideharu Hagiya, Takashi Yorifuji, Toshihiro Koyama, Mitsuru Tsuge, Masato Yashiro, Hirokazu Tsukahara

**Affiliations:** 1 Department of Pediatrics, Okayama University Graduate School of Medicine, Dentistry, and Pharmaceutical Sciences, 2-5-1 Shikata, Okayama, 700-8558 Japan; 2 Department of Pediatrics, Okayama University Hospital, 2-5-1 Shikata, Okayama, 700-8558 Japan; 3 Department of General Medicine, Okayama University Graduate School of Medicine, Dentistry, and Pharmaceutical Science, 2-5-1 Shikata, Okayama, 700-8558 Japan; 4 Department of Epidemiology, Graduate School of Medicine, Dentistry, and Pharmaceutical Sciences, Okayama University, 2-5-1 Shikata, Okayama, 700-8558 Japan; 5 Department of Health Data Science, Graduate School of Medicine, Dentistry, and Pharmaceutical Sciences, Okayama University, 2-5-1 Shikata, Okayama, 700-8558 Japan; 6 Department of Pediatrics Acute Diseases, Okayama University Academic Field of Medicine, Dentistry, and Pharmaceutical Science, 2-5-1 Shikata, Okayama, 700-8558 Japan

**Keywords:** RSV, Surveillance, Google Trends, Epidemiology

## Abstract

**Background:**

The respiratory syncytial virus (RSV) disease burden is significant, especially in infants and children with an underlying disease. Prophylaxis with palivizumab is recommended for these high-risk groups. Early recognition of a RSV epidemic is important for timely administration of palivizumab. We herein aimed to assess the correlation between national surveillance and Google Trends data pertaining to RSV infections in Japan.

**Methods:**

The present, retrospective survey was performed between January 1, 2018 and November 14, 2021 and evaluated the correlation between national surveillance data and Google Trends data. Joinpoint regression was used to identify the points at which changes in trends occurred.

**Results:**

A strong correlation was observed every study year (2018 [*r* = 0.87, *p* < 0.01], 2019 [*r* = 0.83, *p* < 0.01], 2020 [*r* = 0.83, *p* < 0.01], and 2021 [*r* = 0.96, *p* < 0.01]). The change-points in the Google Trends data indicating the start of the RSV epidemic were observed earlier than by sentinel surveillance in 2018 and 2021 and simultaneously with sentinel surveillance in 2019. No epidemic surge was observed in either the Google Trends or the surveillance data from 2020.

**Conclusions:**

Our data suggested that Google Trends has the potential to enable the early identification of RSV epidemics. In countries without a national surveillance system, Google Trends may serve as an alternative early warning system.

**Supplementary Information:**

The online version contains supplementary material available at 10.1186/s12889-022-13899-y.

## Introduction

Respiratory syncytial virus (RSV) is a common cause of acute respiratory tract illness [1–3]. The disease burden is significant, especially in infants and patients with an underlying disease, including premature infants and children with a chronic lung disease, congenital heart failure, immunocompromised status, etc.[1, 2]*.* Prophylactic administration of palivizumab is recommended for these high-risk groups [4]. Japanese national health insurance coverage for palivizumab administration in this population is limited to eight months of the year, usually August to March, during which RSV epidemics normally occur. Recently, RSV epidemics have begun to occur earlier [5] and have become difficult to predict. Therefore, early recognition of a RSV epidemic is crucial to timely and appropriate administration of palivizumab for prophylaxis.

Surveillance systems for respiratory infections, including RSV, vary internationally. Weekly surveillance is the norm in the United States and United Kingdom [6, 7]. In Japan, a sentinel surveillance system at primary care clinics and hospitals contributes to nationwide surveillance by providing weekly updates on the National Institute of Infectious Diseases website [8]. The national surveillance of RSV infections in Japan is reserved only for the pediatric population because RSV mainly affects children. Owing to the development of such national surveillance systems, large RSV epidemics were identified in these countries in 2021 despite the strain on public health resources caused by the coronavirus disease 2019 (COVID-19) pandemic [6, 7, 9, 10]. However, some high-income countries and most middle-income countries still lack a RSV surveillance system and are unable to detect an epidemic early or assess an ongoing epidemic accurately. Concerns about RSV outbreaks are increasing worldwide, calling for a readily accessible method of detection.

Some recent studies reported the utility of search engine query data in predicting a disease trend or an infectious disease epidemic. Google Trends is a tool for exploring a variety of themes pertaining to social and health topics [11], and its data on the influenza virus and COVID-19 were found to correlate with official surveillance data [12–15]. Therefore, we herein aimed to evaluate the correlation between national surveillance and Google Trends data on RSV infections in Japan to assess the utility of Google Trends as a tool for detecting increases in the RSV infection trend.

## Methods

Google Trends data, generated from the total Google search data (https://trends.google.com/trends/?geo=JP), were used as search engine query data. Google Trends data are only available in the form of relative search volume, which is scaled on an index ranging from 0 to 100 (100 is the highest search volume in a given period). The search term, “RS virus” in Japanese (“RS uirusu”) was used to conduct a search in Japan between January 1, 2018, and November 14, 2021. A full-year analysis was conducted to obtain the weekly relative search volume (each year contained 52—53 weeks). The relevant data were collected on November 20, 2021.

The official surveillance data in Japan are reported weekly by the Infectious Disease Surveillance Center at the National Institute of Infectious Diseases [8]. In Japan, data on common infectious diseases, including RSV infections, are collected via sentinel surveillance from about 3,000 pediatric sentinel sites [16]. The data are then expressed as the number of laboratory-confirmed cases per sentinel site and made available to the public via websites after about nine to ten days. In the study period, the surveillance data were available from February 26 (week 9), 2018 to November 7 (week 44), 2021 because the RSV sentinel surveillance system was modified during week 9 in 2018 to report laboratory-confirmed cases per sentinel site rather than the number of actual cases. The raw data are included the supplementary files.

The Spearman rank correlation test was used to compare the Google Trends data with the official surveillance data. Two-sided *p* < 0.05 was considered to indicate statistical significance. Strong, moderate, mild, weak, and no correlation was defined as 0.8–1.0, 0.6–0.8, 0.4–0.6, 0.2–0.4, and 0.0–0.2, respectively. Statistical analysis was performed using EZR (Saitama Medical Center, Jichi Medical University, Saitama, Japan), a graphic user interface for R (The R Foundation for Statistical Computing, Vienna, Austria) [17].

Additionally, to evaluate changes in epidemic trends, the surveillance data and relative search volume in Google Trends were analyzed using the Joinpoint Regression Program, Version 4.9.1.0 (Statistical Research and Applications Branch, National Cancer Institute), which enables the analysis of Joinpoints to identify significant trend changes. We assume that the epidemic curve of RSV infections is usually observed as a single peak each year. Thus, three Joinpoints (change-points) were established to estimate the epidemic trend over the following four periods: period 1) the pre-epidemic phase; period 2) epidemic phase (increasing); period 3) epidemic phase (decreasing); and period 4) post-epidemic phase. This pattern would fail to appear if an epidemic did not occur. The inclination was expressed as weekly percentage changes (WPCs) between change-points with 95% confidence intervals (CI). The present study was approved by the institutional review board of Okayama University Hospital (No. 2111–025).

## Results

**Fig. 1:**
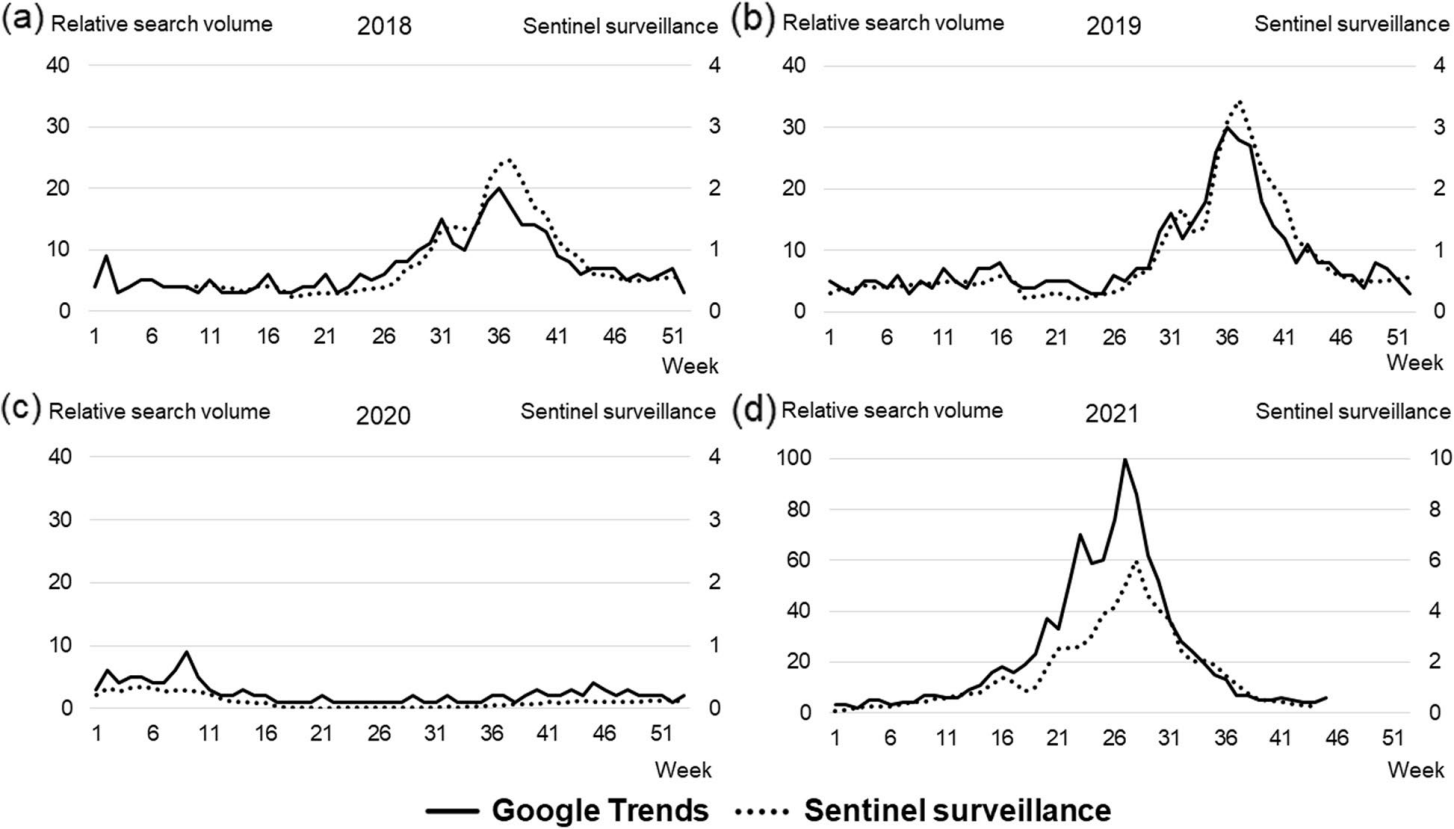
Relative search volume on Google Trends and sentinel surveillance data from 2018 to 2021. Relative search volume on Google Trends and sentinel surveillance data for **a** 2018, **b** 2019, **c** 2020, and **d** 2021. The surveillance data were available from February 26, 2018 to September 12, 2021. The sentinel surveillance data were expressed as the number of laboratory-confirmed cases per sentinel site

**Fig. 2:**
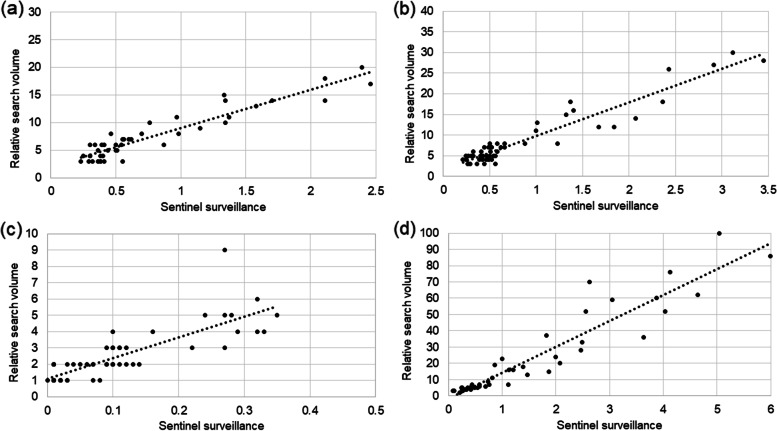
Correlation between sentinel surveillance data and relative search volume on Google Trends per year. The correlation coefficient was 0.87, 0.83, 0.83, and 0.96 for 2018, 2019, 2020, and 2021, respectively. The sentinel surveillance data were expressed as the number of laboratory-confirmed cases per sentinel site

**Fig. 3:**
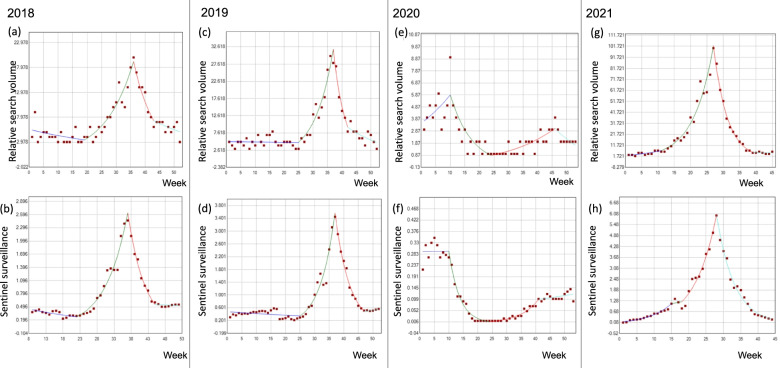
Trend change analysis of relative search volume on Google Trends and sentinel surveillance data between 2018 and 2021 using Joinpoint regression. Three Joinpoints were established and evaluated to determine whether any significant change points occurred between each period. The relative search volume on Google Trends and the sentinel surveillance data for 2018 (**a, b**), 2019 (**c, d**), 2020 (**e, f**), and 2021 (**g, h**) are shown. The blue, green, red, and light blue line indicates period 1, 2, 3, and 4, respectively. In graphs **a**, **b**, **c**, **d**, and **g**, the change- points between the blue and green lines indicate the start of a RSV epidemic. In the graph **h**, the start of a RSV epidemic appears as a change-point between the green and red lines

Figure 1 shows the trends in the relative search volume on Google Trends and the sentinel surveillance data for each year. A strong correlation between these data was observed for every year, namely, 2018 (*r* = 0.87, *p* < 0.01), 2019 (*r* = 0.83, *p* < 0.01), 2020 (*r* = 0.83, *p* < 0.01), and 2021 (*r* = 0.96, *p* < 0.01) (Fig. 2). The rising curve in the Google Trends data preceded that of the sentinel surveillance data in 2018, 2019, and 2021 (Figs. 1a, b, d). No epidemic surge was observed on either Google Trends or the surveillance data in 2020 while a large epidemic surge was observed in 2021. Table 1 and Fig. 3 show the results of Joinpoint trend analysis of the relative search volume on Google Trends and the sentinel surveillance data. The change-points in period 2 suggesting the onset of a RSV epidemic were observed at week 19 on Google Trends and at week 22 in the sentinel surveillance data for 2018. The change- points in 2019 showed the same pattern at week 25. The change-points in period 2 in 2020 appeared during week 10 in both datasets, but the epidemic peak was not observed this season as already shown in (Fig. 1c). In 2021, as in 2018, the Google Trends data showed a change-point at week 11, or earlier than the sentinel surveillance data at week 15 or 18. Joinpoint analysis of the surveillance data from 2021 revealed that the increasing phase corresponded to period 3 as shown in (Fig. 3h) rather than to period 2.

**Table 1:** Trend changes in sentinel surveillance data and relative search volume on Google Trends for RSV between 2018 and 2021

	Year	Period 1 (week)	WPC (95% CI)	Period 2 (week)	WPC (95% CI)	Period 3 (week)	WPC (95% CI)	Period 4 (week)	WPC (95% CI)
Google Trends	2018	1–18	-2.6 (-5.0 – 0.0)	19–35	10.7^a^ (8.2 – 13.3)	36–42	-12.9^a^ (-19.9 – -5.4)	43–52	-4 (-9.9 – 2.4)
Sentinel surveillance	2018	9–21	-3.6^a^ (-6.1 – -1.0)	22–36	16.4^a^ (14.6 – 18.2)	37–44	-17.8^a^ (-20.7 – -14.8)	45–52	-0.5 (-5.6 – 4.8)
Google Trends	2019	1–24	-0.2 (-1.9 – 1.6)	25–36	17.0^a^ (13.1 – 21.0)	37–41	-21.9^a^ (-31.6 – -10.8)	42–52	-6.3^a^ (-1.2 – -2.5)
Sentinel surveillance	2019	1–24	-1.4 (-3.1 – 0.4)	25–36	21.6^a^ (17.8 – 25.6)	37–46	-18.1^a^ (-21.2 – -14.8)	47–52	2.6 (-11.5 – 18.8)
Google Trends	2020	1–9	5.2 (-0.1 – 10.8)	10–23	-12.8^a^ (-16.1 – -9.3)	24–45	6.0^a^ (3.6 – 8.4)	46–53	-8.2 (-16.9 – 1.5)
Sentinel surveillance	2020	1–9	0 (-2.6 – 2.8)	10–23	-23.7^a^ (-26.9 – -20.4)	24–40	17.1^a^ (12.8 – 21.6)	41–53	1.7 (-1.1 – 4.5)
Google Trends	2021	1–10	9.6^a^ (0.9 – 19.0)	11–26	18.2^a^ (15.9 – 20.5)	27–38	-21.6^a^ (-24.0 – -19.2)	39–45	-1.9 (-16.5 – -15.3)
Sentinel surveillance	2021	1–14	16.8^a^ (12.6 – 21.1)	15–17	3.7 (-29.1 – 51.6)	18–27	16.6^a^ (13.8 – 19.5)	28–44	-16.4 (18.8 – -14.0)

^a^Statistically significant

*WPC* Weekly percentage change, *95% CI* 95% confidence interval

## Discussion

The present study revealed a strong correlation between the sentinel surveillance data and relative search volume on Google Trends. The strength of the Internet search engine query data has significant implications for real-world public health interests. Additionally, our findings suggested that Google Trends may have the potential to enable early detection of a RSV epidemic even if a national surveillance system is unavailable.

The analysis of Google Trends data pertaining to infectious diseases was initially used to determine its utility in detecting the influenza A(H1N1)2009 pandemic, in which it demonstrated an ability to forecast influenza disease activity [12]. The Google Trends data for estimating the activity of the influenza virus, dubbed “Google Flu Trends”, showed a favorable correlation in the United States and Europe [13, 15, 18, 19]. Recently, the utility of Google Trends for describing RSV activity in the United States was also evaluated [20, 21]. Although this has been done only in the United Sates thus far, our findings indicated that it may be applicable to other nations as well. Furthermore, our analysis suggested that Google Trends might be capable of detecting an increase in the RSV infection trend simultaneously with or even before the national surveillance system. Further evaluation in other countries or regions is needed. Google Trends is a readily available tool that can be used with great effect to advise the public health sector of infection risks. For countries that do not have a nationwide surveillance or alert system, Google Trends may serve as a useful, alternative warning system provided that the Internet penetration rate is at level comparable with that of the nations discussed.

Early recognition of a RSV epidemic is important because it enables timely palivizumab administration to prevent infections in high-risk patients. The RSV epidemic was observed earlier in 2021 than in 2018 or 2019. This anomaly may be explained by the fact that no epidemic surge occurred in the preceding year. In situations such as that seen in 2021, early prophylaxis with palivizumab should be considered. In Japan, the Infectious Disease Surveillance Center issued an alert concerning a RSV epidemic in week 18 in 2021 [22]. Our trend analysis using Joinpoint regression indicated that the RSV epidemic started earlier, at around week 11. If the Google Trends database had been used to monitor the RSV trend, a timelier warning might have been issued to the public health sector.

Although Google Trends analysis has important implications for early epidemic detection, the peak of the epidemic curve in Google Trends was higher than in the surveillance data for 2021. We suspected that this discrepancy was affected by the public’s interest in the RSV epidemic, which the search volume on Google Trends reflects. Additionally, in 2020, a small peak was observed in the Google Trends data at week 9 while no peak was observed in the surveillance data. A small peak of this sort, which was apparently unrelated to any disease trend, may create the false impression that an epidemic is imminent. No epidemic surge in the sentinel surveillance was observed in 2020 owing to the governmental strategy for dealing with the COVID-19 pandemic (declaration of emergency status), including sheltering at home and closing schools. [23–25]. Moreover, topics of public interest, such as the announcement of a new drug or vaccine for RSV, will likely affect the search volume on Google Trends, undermining the reliability of the findings. Thus, Google Trend analysis is not an infallible method of predicting an infectious disease epidemic. Further studies are needed to evaluate the advantages and disadvantages of Internet search engine query data pertaining to other diseases and in other countries.

The present study had some limitations. First, the generalizability of the findings to other countries and regions was not evaluated. However, previous studies of Google Flu Trends demonstrated the service’s utility in the United States and Europe [10, 11, 13, 16, 17]; we may therefore expect a similar utility in predicting RSV trends. Second, we were able to obtain only a “relative” search volume because the “actual” search volume of Google Trends data is not available to the public. If the total number of Internet searches was very small, the results of an analysis of Google Trends data might become susceptible to over- or underestimation.

In conclusion, our study found a strong correlation between the relative search volume on Google Trends and sentinel surveillance data on RSV infections. Additionally, the Google Trends database was found to be able to detect an increasing trend in RSV infections simultaneously with or even before the national surveillance system. With its wide availability and user-friendly interface, Google Trends will likely gain more attention for its utility as a surveillance system for infectious diseases even among patients and their guardians.

## Supplementary Information

**Additional file 1.** Relative search volume on Google Trends and sentinel surveillance data for 2018, 2019, 2020, and 2021 were shown in the supplementary data. The Google Trends data and surveillance data were available from January 1, 2018, to November 14, 2021, and from February 26, 2018, to September 12, 2021. The sentinel surveillance data were expressed as the number of laboratory-confirmed cases per sentinel site. NA: not available.

## Data Availability

The data for this study are available at https://trends.google.co.jp/trends/explore?date=2018-01-01%202021-11-14&geo=JP&q=%2Fm%2F02f84_ and in the Infectious Diseases Weekly Report of the National Institute of Infectious Diseases (https://www.niid.go.jp/niid/ja/data/10762-idwr-sokuho-data-j-2144.html). The raw data are included in the supplementary files.
